# Application of sequential haplotype scan methods to case-control data

**DOI:** 10.1186/1753-6561-1-s1-s21

**Published:** 2007-12-18

**Authors:** Zhaoxia Yu, Daniel J Schaid

**Affiliations:** 1 Division of Biostatistics, Department of Health Sciences Research, Mayo Clinic College of Medicine, Harwick 7, 200 First Street Southwest, Rochester, Minnesota 55905, USA

## Abstract

Haplotype association analysis based on arbitrarily chosen markers might lower statistical power because of the larger number of degrees of freedom caused by irrelevant makers.

On the other hand, an exhaustive search for all possible combinations of markers for haplotype analysis is computationally expensive for genome-wide association analysis.

To improve power, we applied our recently developed sequential haplotype scan method to case-control data for rheumatoid arthritis, including the *PTPN22 *candidate gene on chromosome 1p and the association mapping data on chromosome 18q, from the Genetic Analysis Workshop 15. The results showed that our new approach is at least as powerful as the traditional single-locus analysis and sometimes can be more powerful.

## Background

Haplotype association analysis may provide greater power than single-locus analysis because it naturally combines information from multiple loci. In practice, many investigators use a sliding window method with a arbitrarily chosen window size. However, statistical power can be diminished because of the extra degrees of freedom contributed by irrelevant genetic markers. Therefore, choosing a proper set of markers for haplotype analysis is essential to improve statistical power. A properly chosen set of markers for haplotype analysis should only contain markers that contribute considerable amount of information to disease status. Cheng et al. [1] used an exhaustive search of all possible sets of adjacent markers that flank a marker. More recently, Bahlo et al. [2] introduced a summary statistic that sums over a series of nested chi-square statistics. Although these approaches avoid arbitrarily choosing a window size, they are either computationally expensive or might only provide greater power than the traditional single-locus method in some specific scenarios.

Recently, we proposed a sequential haplotype scan approach [3]. Using this method, we add markers close to each other in a sequential manner: a marker is added if it provides extra information for detecting the haplotype association with disease, conditional on current hapltotypes. This is assessed by the Mantel-Haenszel (MH) test [4]. Our previous study shows that, compared with the traditional single-locus analysis, out new methods can either have minor loss or substantial gain in power under a variety of genetic models [3]. We applied our method to two data sets provided by the North American Rheumatoid Arthritis Consortium (NARAC): the *PTPN22 *candidate gene data and the association mapping data.

## Methods

### Statistical method

For now, assume haplotype phase is known. Therefore, for a sample with *N *subjects, there are 2*N *haplotypes. Let *Y *denote disease status for each of the 2*N *haplotypes, with values of 0 and 1 according to whether a haplotype came from a control or case, respectively. Let *X *denote the 2*N *alleles of a new single-nucleotide polymorphism (SNP) we wish to add to the current set of haplotypes, with values 0 and 1 according to whether an allele is common or rare, respectively. Let *H *be the number of distinguishable current haplotypes. We create *H *strata according to the current set of haplotypes, and use the MH procedure to test the association of *X *and *Y*, conditional on the current haplotypes. For the *h*^th ^stratum, let *n*_*ijh *_denote the number of haplotypes with *X *= *i*, and *Y *= *j*. It is well known that conditional on fixed row and column margins, the entry *n*_11*h *_has a hypergeometric distribution. Under the null hypothesis that *X *and *Y *are not associated in any stratum, the MH statistic,

$$MH=\frac{{\left[\sum_{h}\left(n_{11h}-E(n_{11h})\right)\right]}^{2}}{\sum_{h}Var(n_{11h})}$$

has an asymptotic chi-square distribution with one degree of freedom, where

$$\begin{array}{cc} \mu_{11h}=E(n_{11h})=\frac{n_{1+h}n_{+1h}}{n_{++h}}, & Var(n_{11h})=\frac{n_{1+h}n_{0+h}n_{+1h}n_{+0h}}{n_{++h}^{2}(n_{++h}-1)}. \end{array}$$

By applying the MH approach sequentially, we can decide which markers should be added to a variable length haplotype. When the haplotype phase is unknown, for each subject the posterior probabilities of all possible haplotype pairs were estimated by the expectation maximization (EM) algorithm using haplo.em [5]. Then the estimated haplotypes were used in a weighted fashion. In this situation, *n*_*ijh *_is the expected count on the basis of the sum of posterior probabilities of all estimated haplotypes with *X *= *i *and *Y *= *j *in the *h*^th ^stratum, which could be a fraction instead of an integer.

When scanning SNP *X*_0 _at position *x*, we examined SNPs close to it on both sides to determine if they provide additional information for association. The two alleles 0 and 1 of SNP *X*_0 _separate the sample of alleles into two strata: those with allele 1 and those with allele 0. We first examined whether at least one of the nearest SNPs on each side (left and right) of *X*_0 _provides information for association, conditional on *X*_0_. If at least one of them offers substantially additional information, we combined *X*_0 _with the SNP(s) into a multilocus haplotype variable and test if the second nearest marker on each side of *X*_0 _should be combined. The process is continued until no SNP should be added. More details about the algorithm can be found in Yu and Schaid [3]. Once the sequential search procedure ends, two test statistics are then calculated: 1) A -log_10_(*p*-value) for the haplotype-based chi-square test for the contingency table of the haplotypes constructed from all makers in the sequentially chosen SNPs and disease status. Denote this statistic by $\chi_{H}^{2}$(*x*). 2) A -log10(*p*-value) for the sum of conditional chi-square statistics, Sum(*x*). Because conditioning creates independent chi-square statistics, Sum(*x*) has an asymptotic chi-square distribution with the degrees of freedom equal to the number of variables combined. Denote this statistic by $\chi_{S}^{2}$(*x*).

Thus, for the marker at physical position *x*, three statistics are calculated: 1) the traditional single-marker chi-square statistic $\chi_{0}^{2}$(*x*) that uses the marker being scanned only, 2) the sequential haplotype statistic $\chi_{H}^{2}$(*x*), and 3) the sequential summary statistic $\chi_{S}^{2}$(*x*). With permutation of the disease status, a pointwise *p*-value is defined as the percentage of times that the permuted statistic is larger than the observed statistic at a position. On the other hand, when we examine regional *p*-values, we used the maximum of the statistics across the whole region as the test statistic and define *p*-values correspondingly. Both the pointwise and regional *p*-values were calculated.

### Data

The data from the *PTPN22 *gene [6] contain both case siblings and unrelated controls. To create unrelated case-control data, one sib from each case family was randomly chosen. When analyzing the data, we assumed that the confirmed variant R620W was not observed, i.e., we used its surrogate markers (12 SNPs). To evaluate the pointwise and regional associations with disease status, one million permutations were used. The NARAC association mapping data consist of 2300 SNPs across a 10-Mb region on chromosome 18q. The data were collected from 460 unrelated cases and 460 unrelated controls. Two individuals with 5% or more missing SNPs were removed. After dropping markers with minor allele frequency less than 0.05 or *p*-value of Hardy-Weinberg equilibrium test less than 0.01, 2186 SNPs were used in the analysis. The pointwise and regional associations were evaluated using 10,000 permutations.

## Results

**Figure 1:**
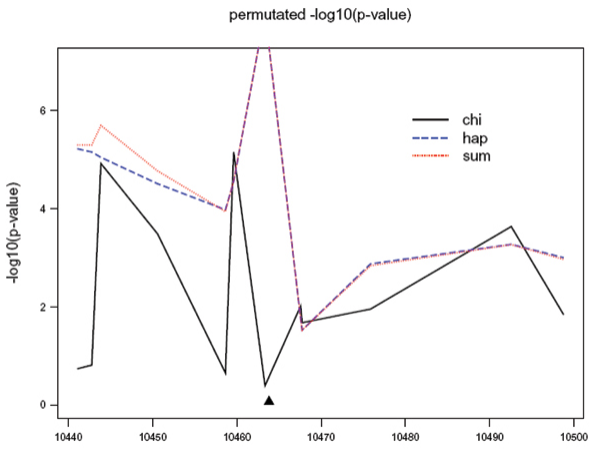
**Results for the PTPN22 data**. The triangle indicates the physical position of the G620W variant, which was assumed not measured in our analysis. The *p*-values of $\chi_{S}^{2}$ (*x*) and $\chi_{H}^{2}$ (*x*) are truncated at the seventh SNP because their permutated *p*-values are zero based on one million permutations.

The pointwise *p*-values on the -log10 scale for the *PTPN22 *data are plotted in Figure 1. The *p*-values of the sequential haplotype method and the sequential summary method show stronger association than those of the single-locus method at several places. It is interesting to observe that the *p*-value of the nearest marker to R620W is not significant at the 0.05 level using the single-locus method but the *p*-values based on the two sequential methods, which combine one SNP on the left and two SNPs on the right, are less than 10^-6^. The regional *p*-values using the single-locus, the sequential haplotype, and the sequential summary methods are 7.8 × 10^-5^, <10^-6^, and <10^-6^, respectively. Therefore, compared to the single-locus test, tests based on the two sequential methods we proposed provide stronger evidence for the association between this region and disease status.

For the NARAC association mapping data, the regional *p*-values for the single-locus, the sequential haplotype, and the sequential summary methods are 0.328, 0.039, and 0.089, respectively. Our sequential approach found several sets of SNPs that show strong significance. For example, a combination identified by our sequential approach, SNPs at physical positions 4820404, 4845230, and 4850927, provide a four-degree-of-freedom chi-square statistic of value 25.29. The corresponding permutated *p*-value is less than <10^-4 ^(the asymptotic *p*-value is 4.4 × 10^-5^). Although the single-locus method does not show statistically significant evidence for association, the two sequential methods provide weak regional association.

## Conclusion

We applied our recently developed sequential haplotype scan methods to two of the rheumatoid arthritis case-control data sets from GAW15. For the association mapping data on chromosome 18q, our two sequential methods provide weak regional evidence for association while the single-locus method does not. The results for *PTPN22 *indicate that our new approach might be more powerful than the traditional single-locus analysis on both pointwise and regional levels. This sequential haplotype scan procedure uses the MH statistic to examine whether additional markers are informative for association, conditional on haplotypes constructed from sequentially chosen markers. The MH statistic has a large-sample chi-square distribution with one degree of freedom and has been proven to be equivalent to the efficient score statistic for logistic regression [7]. One advantage of using the MH approach is that we can compute the test statistic rapidly without iteratively estimating parameters of logistic regressions. The MH test is also robust for tables with large number of small strata and zero cell entries [8] and it is the uniformly most powerful unbiased test [9]. By adding markers sequentially based on the MH procedure and scanning all makers, our sequential haplotype scan methods can improve the statistical power in detecting the association of haplotypes with disease.

One limitation of our procedure is that it cannot detect effects of haplotypes constructed from markers that are far apart. Indeed, this is one of the most difficult situations to detect for most methods that have been developed to date. In a candidate gene region with a relatively small number of markers, we may modify the stopping rule of the sequential scan procedure so that a more comprehensive analysis can be performed. Extensions of our sequential scan method with strategies allowing for testing markers that are not physically close to each other might be helpful and will be studied in the future.

## Competing interests

The author(s) declare that they have no competing interests.
